# Facial lesions in piglets with intact or grinded teeth

**DOI:** 10.1186/1751-0147-54-23

**Published:** 2012-04-05

**Authors:** Monica Hansson, Nils Lundeheim

**Affiliations:** 1 Department of Animal Breeding and Genetics, Swedish University of Agricultural Sciences, P.O. Box 7023, SE-750 07 Uppsala, Sweden

## Abstract

**Background:**

Piglets are born with eight sharp teeth that during nursing can cause facial lesions on littermates and teat lesions on the sow. Teeth grinding in piglets is therefore often practiced to reduce these lesions. The aim of this study was to assess the consequences of grinding piglet teeth in regard to the occurrence of lesions.

In this study the piglets' teeth were grinded in 28 litters, and in 36 litters the piglets' teeth were kept intact. Twice, one time during the first week and one time during the second week after birth facial lesions of the piglets were scored and the teats of the sows were examined for lesions. The facial lesion score accounted for the amount and severity of lesions. The individual observations on piglets in the litter were synthesized in a litter facial lesion score.

**Findings:**

69.8% and 43.5% of the piglets had facial lesions in week 1 and week 2 respectively. The effect of treatment was not significant on litter facial lesion score. The litter facial lesion score was higher in week 1 than in week 2 (*p *< 0.001) and higher in large litters (*p *= 0.003) than in small litters. Mortality between week 1 and week 2 was higher in litters with intact teeth (*p *= 0.02). Sow teat lesions only occurred if litters had intact teeth.

**Conclusions:**

According to our results teeth grinding is only justifiable in large litters.

## Findings

Piglets are born with 28 teeth; three incisors, one canine tooth and three premolars per jaw half [1]. The teeth are used by the piglets to establish a teat order during the first hours after birth [2] and subsequently to defend their specific teat [3]. The canine teeth and the third pairs of incisors are sharp and slightly angled outwards (laterally) from the jaw [4]. They can cause facial lesions on littermates, and teat and udder lesions on the sow. To reduce the incidence of such lesions sharp teeth are often clipped or grinded in commercial pig production.

According to EU legislation (Directive, 2001/93/EC), teeth clipping and grinding are only allowed when "there is evidence that injuries to sows' teats or to other pigs' ears or tails have occurred". In Sweden teeth clipping is forbidden since 2008 (SJVFS 2009:85, D8), but grinding is permitted if it can be demonstrated that lesions on the littermates or the sow have been caused by intact teeth. Grinding is only allowed during the piglets first week of life.

Clipping and grinding may lead to reduced animal welfare, both due to stress during handling [5,6] and the risk of infection and teeth damage following teeth clipping and grinding [7-10]. The dental pulps are provided with nerve fibres and blood vessels, thus damage to the pulp cause pain. Bacteria can enter and cause arthritis if an opening of the pulp cavity occurs. In addition, if a pulp infection occurs, bacteria can enter the mammary gland of the sow trough the teat canal during nursing and may cause mastitis [11].

The aim of this study was to assess the consequences of grinding piglets' teeth on the incidence of facial lesions in piglets and of lesions on the sow teat.

The study was approved by the Ethical Committee for Animal Experiments, Uppsala, Sweden (reference number C 6/11). It was conducted during five weeks in February - March 2011 in one piglet-producing herd in the south-central part of Sweden. The production was batch-wise with one week between farrowing. The sows were Landrace x Yorkshire crosses which were inseminated with Hampshire or Duroc AI-boars. The sows farrowed in individual pens and were loose-housed.

The study included 64 litters (777 piglets) from four farrowing batches among which 28 litters had their piglets teeth-grinded. Herdsmen grinded the eight sharp teeth during the piglet first day of life, using a mechanical grinder (Dremel^®^, battery driven).

Piglet facial lesions were examined twice, once during their first and once during the second week after birth. The lesions were scored according to Fraser [3] on a scale from 0-3 where 0 = no lesions, 1 = minor (< 5 lesions), 2 = medium and 3 = all surface covered with lesions. Lesions on teats of sows were scored on the same occasions. These lesions were scored from 0-3 where 0 = no lesions, 1 = lesions on less than 50% of teats, 2 = lesions on more than 50% of teats, and 3 = lesions on all teats of the udder.

The technician scoring the lesions did not know which litters had their piglets teeth-grinded. Additional information was registered, including farrowing date, parity number and if cross-fostering was used.

Statistical analyze were performed using SAS Software 9.2 (SAS Institute Inc., Cary, NC). Normal distribution was checked using proc univariate. The scoring of individual facial lesions in piglets was synthesized in a litter facial lesion score corresponding to the incidence of facial lesions in the litter. For every piglet in the litter the scoring was transformed, 0 → 0, 1 → 2, 2 → 4 and 3 → 7 (7 instead of 6 was used to better reflect the negative impact on animal welfare) and total score of the litter was then divided by the number of piglets in the litter at the examination occasion. The litter facial lesion score ranged from 0 to 7.

The litter facial lesion score was analysed using analysis of variance (proc mixed). The statistical model included the fixed effects of treatment (2 classes, teeth intact or teeth grinded), examination occasion (week 1 and week 2), cross-fostering (yes, no), and the random effect of sow identity (64 classes) nested within treatment to account for repeated score on week 1 and week 2. Litter size was included in the model as a continuous covariate. The interaction between treatment and examination occasion was almost significant (*p *= 0.08) and was therefore included in the model. Parity number did not have a significant effect on the litter facial lesion score and was therefore not included in the statistical model.

In the analysis at sow scale teat lesions were treated as a 0/1-variable, no lesions (score 0) or lesions (score 1 to 3 grouped together). Piglet mortality between week 1 and week 2 was also treated as a 0/1-variable, according to absence or presence of mortality in the litter.

Mortality between week 1 and 2 was analysed using proc glimmix (binomial distribution) and the statistical model included the fixed effects of treatment and cross-fostering. Litter size was included in the model as a continuous covariate. P-values ≤ 0.05 were regarded as significant.

### Descriptive statistics

**Figure 1:**
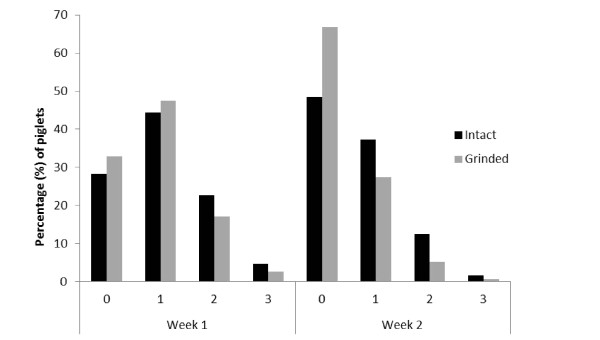
**Scoring of facial lesions, by week and treatment**. Piglets with grinded teeth had less severe (score 2-3) facial lesions than piglets with intact teeth, both in week 1 and 2.

The results of the study showed that the incidence of individual facial lesions (score 1-3) affected 69.8% of the piglets in week 1, and 43.5% of the piglets in week 2. The distribution of facial lesions for each treatment in week 1 and 2 is shown in Figure 1.

In both week 1 and week 2 there was a higher proportion of piglets with lesion score 2 and 3 in litters with intact teeth than in litters with grinded teeth. The proportion of piglets free from facial lesions was higher when teeth were grinded, in both week 1 and week 2. The incidence of severe facial lesions (score 2-3) decreased between week 1 and week 2 for both treatments.

**Figure 2:**
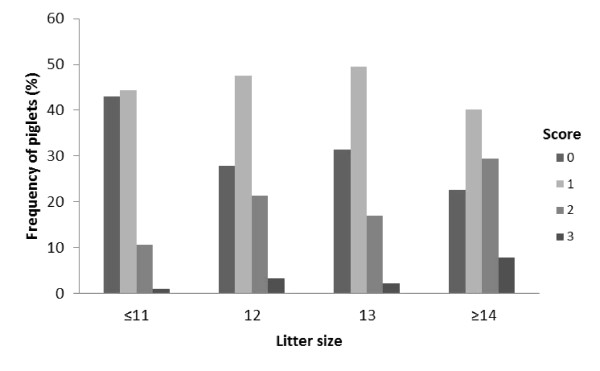
**Litter facial scores on week 1 according to litter size**. The effect of litter size on facial lesion score was significant (*p *= 0.003).

**Figure 3:**
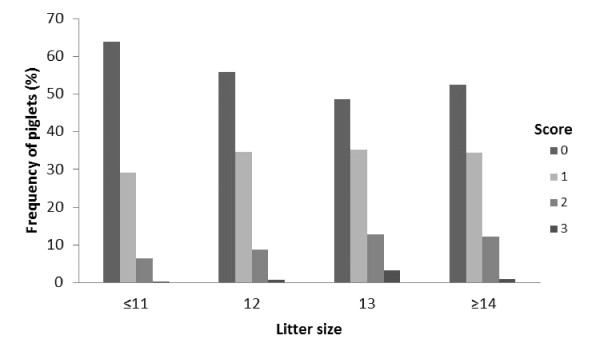
**Litter facial scores on week 2 according to litter size**. The effect of litter size on facial lesion score was significant (*p *= 0.003).

The proportion of piglets with facial lesions was higher in larger litters, in both week 1 and week 2 (Figure 2 and 3). The proportion of piglets with scores 2 and 3 increased with litter size. Table 1 show how many litters that were present in each litter size group.

**Table 1:** Number of litters by litter size group, week and treatment

Litter size	Week 1		Week 2	
	**Intact**	**Grinded**	**Intact**	**Grinded**

≤ 11	9	6	14	11
12	10	13	11	9
13	9	5	8	4
≥ 14	8	4	3	4

The average litter size in litters with intact teeth in week 1 was higher compared to that of litters with grinded teeth. However in week 2 it was the opposite (Table 2).

**Table 2:** Mean and SD (standard deviation) for litter size, by week and treatment

	**Litter size**		**Litter size**		
	
	**Week 1**	**SD**	**Week 2**	**SD**	**Mortality**
	
Intact	12.3	2.2	11.5	1.9	0.8^a^
Grinded	12.0	1.1	11.6	1.8	0.4^b^

There were no differences in litter sizes between the treatment groups but the mortality between week 1 and 2 was significantly (*p *= 0.02) higher in litters with intact teeth than in litters with grinded teeth. Means with different letters indicate significant difference (*p *< 0.05)

Table 3 shows that the highest mean for the litter facial lesion score was found in week 1 in litters with intact teeth, and the lowest in week 2 in litters with grinded teeth.

**Table 3:** Least square means and SE (standard error) for litter facial lesion score, by week and treatment

	**Week 1**	**SE**	**Week 2**	**SE**
	
Intact	2.00	2.00	1.28	1.23
Grinded	1.79	1.79	0.79	0.79

Both in week 1 and 2, facial lesion score was higher in litters with intact teeth than in litters with grinded teeth. However, the difference was not significant

### Analyses of variance

Treatment did not have a significant effect on piglet litter facial lesion score. However, litter size had a significant effect on litter facial lesion score (*p *= 0.003); one more piglet in the litter increased the litter score of 0.16 units. Litter facial lesion score increased with litter size.

Examination occasion had a significant effect on the litter facial lesion score (*p *< 0.001). The litter facial lesion score in week 1 was 0.86 units higher than in week 2. In litters with cross-fostering, the litter facial lesion score was lower (1.28, SE 0.16) than in litters without cross-fostering (1.65, SE 1.14) (*p *= 0.08).

Piglet mortality was observed between week 1 and week 2 in 46% of the litters with intact teeth, and 25% in litters with grinded teeth. This difference was significant (*p *= 0.02).

The number of sows with teat lesions was too low to be analysed statistically. Only three sows had teat lesions in week 1 and nine in week 2. All the sows with lesions had piglets with intact teeth.

In conclusion, this study did not show any significant benefits of grinding the teeth of piglets in regard to litter facial lesion score in the piglets or teat lesions on sows. The majority of facial lesions were scored as moderate and already in week 2 the amount of lesions and the severity had decreased. Litter size had a significant effect on the incidence of facial lesions; therefore, if possible, cross-fostering to decrease the number of piglets might be preferable instead of grinding their teeth.

Teeth grinding involves stress for the piglet and can cause painful teeth damage. These consequences should be kept in mind before teeth grinding is performed. Teeth grinding is only justifiable in large litters with more than 14 piglets.

## Competing interests

The authors declare that they have no competing interests.

## Authors' contributions

MH applied for funding of the study, planned the design of the study, performed the field study, analysed data and drafted the manuscript. NL assisted MH in the statistical analyses and assisted to draft the manuscript. Both authors read and approved the final manuscript.
